# Biomedical utility of zinc and copper mediated cerium oxide nanoparticles

**DOI:** 10.1039/d6ra02316a

**Published:** 2026-04-30

**Authors:** Samantha Stoltz, Craig J. Neal, Elayaraja Kolanthai, Yifei Fu, Sudipta Seal

**Affiliations:** a Advanced Materials Processing and Analysis Center, Department of Materials Science and Engineering, Nanoscience Technology Center, Biionix Cluster, College of Medicine, University of Central Florida Orlando Florida USA Sudipta.seal@ucf.edu

## Abstract

Breast cancer remains a leading cause of cancer-related death worldwide, motivating the development of nanobiomaterials that exploit tumour-associated redox vulnerabilities while limiting systemic toxicity. Cerium oxide nanoparticles (CeO_2_, CNPs) are promising in this context due to their reversible Ce^3+^/Ce^4+^ redox cycling and oxygen vacancy mediated enzyme-mimetic activity, which enables microenvironment dependent modulation of reactive oxygen species. However, defect driven catalytic plasticity complicates predictive design for biological applications. Here, Zn- and Cu-doped CNPs (5–20 mol%) were synthesized *via* forced hydrolysis and characterized using XPS, UV-Vis spectroscopy, XRD, TEM, dynamic light scattering, and zeta potential analysis, confirming fluorite structure with crystallite sizes of ∼4–7 nm and no secondary crystalline phases detectable by XRD, consistent with successful dopant incorporation. XPS analysis revealed dopant dependent differences in surface redox chemistry, with Zn- and Cu-doped CNPs exhibiting distinct Ce^3+^ fractions and redox active surface states. Enzyme-mimetic assays showed that Zn-doped CNPs substantially enhanced superoxide dismutase-like activity at low dopant levels while suppressing catalase-like activity, whereas Cu-doped CNPs significantly increased catalase-like activity at higher dopant loadings, indicating distinct catalytic fingerprints at the nano–bio interface. Normal human unbillical vein endothelial cells (HUVECs) exhibited an increase in cell number at low concentration of CNPs and doped nanoparticles, whereas Zn and Cu-doped CNPs showed significant reduction in cell number at higher concentrations. Collectively, these results demonstrate dopant-gated control of surface redox states as a strategy to tune the catalytic behavior of nanoceria and highlight the potential of doped CNPs as programmable redox-active nanobiomaterials with preferential *in vitro* cytotoxicity toward cancer cells.

## Abstract 2

(Copper, Zinc) Metal (oxide)-modified cerium oxide nanoparticles promote cell survival *via* redox-mediated surface reactions *in vitro* (HUVEC); while producing a substantial pro-oxidant, cytotoxic effect in a (MCF-7 breast cancer) cancer model.

## Graphical abstract

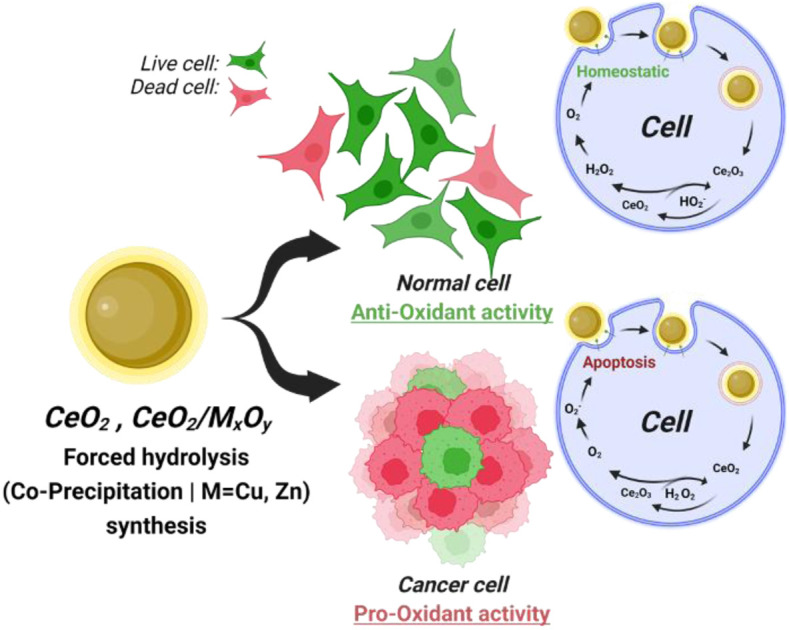

## 1. Introduction

Cancer continues to be a leading cause of death worldwide, despite significant advances in early detection, molecular diagnostics, and therapeutic development.^1,2^ Conventional methods of intervention and treatment such as surgery, radiation therapy, and chemotherapy, continue to dominate clinical practice. However, these approaches are frequently associated with toxicity, acquired resistance, systemic side effects, and reduced life expectancy for patients.^3–7^ Therefore, development of therapeutic strategies that exploit the intrinsic vulnerabilities of malignant cells while minimizing damage to surrounding healthy tissue has been increasingly prioritized.^3–6,8^

Dysregulated oxidative stress is an intrinsic vulnerability that provides a hospitable environment for cancer cells. Cancer cells thrive and replicate in a state of oxidative stress. Elevated reactive oxygen species (ROS) contribute to proliferative signalling, genomic instability, angiogenesis, and metastatic progression, while simultaneously rendering malignant cells more susceptible to further oxidative stress.^9–13^ This positive feedback of excessive ROS accumulation can overwhelm adaptive antioxidant defences, pushing malignant cells beyond a tolerable redox threshold.^14^ This imbalance creates a narrow but potentially exploitable therapeutic window. Thus, an increase in ROS may preferentially induce cancer cell death while normal cells retain sufficient antioxidant capacity to preserve redox homeostasis.^9–12,15^ Despite this conceptual appeal, medical translation of ROS-based therapeutic strategies remains challenging due to limited selectivity, heterogeneous tumour microenvironments, and off-target toxicity.^4,10^

Nanomaterials offer a versatile platform for addressing the limitations of ROS-based therapeutic strategies. At the nanoscale, materials exhibit physicochemical properties that differ from those of bulk materials, including high surface-to-volume ratios, tuneable surface chemistry, and size, with differing biological responses.^3,4,8,16,17^ These properties enable engineered interactions with intracellular compartments, redox pathways, and signalling networks, supporting applications in imaging, drug delivery, radio sensitization, and direct redox modulation.^3,4,8,13,18^

Among inorganic nanomaterials, cerium oxide nanoparticles (CNPs, nanoceria) exhibit unique properties due to their intrinsic redox activity and adaptive catalytic behavior.^7,8,19–21^ Thus, extensive studies on CNPs have led to the discovery of their ability to dynamically modulate oxidative stress through reversible cycling between Ce^3+^ and Ce^4+^ oxidation states, in addition to the formation and annihilation of oxygen vacancies that govern surface reactivity.^8,19,21–24^ The redox plasticity enables nanoceria to function as a context-dependent nanozyme that exhibits catalase-like, superoxide dismutase (SOD)-like, or peroxidase (POD)-like activity depending on the surface chemistry and biological conditions.^8,20–23,25,26^

The biological behaviour of nanoceria is not intrinsic or fixed, but highly context dependent. Cellular responses vary with particle size, defect density, surface charge, hydration state, redox status of the surrounding microenvironment, cell type, and metabolic state.^20,26–28^ These factors underscore that nanoceria does not act as a conventional antioxidant or cytotoxin, but rather as a redox-modulating material whose biological effects emerge from interactions between nanoparticle surface chemistry and cellular regulatory mechanisms.^8,19,21^

The microenvironment of a tumour has been shown to further amplify the complexity of nanoceria's biological behaviour by producing chronic oxidative stress and altered redox signalling. These conditions can sensitize cancer cells to additional oxidative stress while allowing normal cells to maintain redox resilience.^8,28,29^ This differential vulnerability provides a mechanistic basis for the selective activity reported for certain redox-active nanomaterials, including nanoceria based systems, when appropriately engineered.^9,10^

Transition-metal doping has emerged as an effective strategy to engineer nanoceria surface chemistry by modulating oxygen vacancy formation, charge compensation, and redox-state stabilization.^22,23,28,30,31^ Copper-doped CNPs, in particular, have demonstrated enhanced redox activity and pro-oxidant behaviour in cancer-relevant models. This is attributed to the presence of multivalent Cu^+^/Cu^2+^ surface states and their interaction with ceria defect chemistry.^12,13,32^ Similarly, zinc-containing nanomaterials, including ZnO and Zn-doped ceria, have shown context-dependent cytotoxicity and favourable biocompatibility. Thereby making zinc another attractive dopant for tuning redox behaviour. Contrastingly, Zn-containing nanoparticles have exhibited toxicity at higher doses in relevant studies.^16,33–37^ Comparative studies further indicate that nanoceria-mediated redox modulation in cancer cells is highly dependent on formulation, concentration, and cellular context.^3,4,7,13,16,32–35,38^ Despite these advances, direct comparisons of different dopant identities and dopant concentrations under identical synthesis, physicochemical characterization, and biological testing conditions remain limited. In particular, the relationship between dopant-engineered catalytic bias and downstream cellular responses has not been fully defined in systems designed to evaluate cancer cytotoxicity and normal cell compatibility simultaneously.^14,20,34,36,38,39^

In this study, we prepared varying mol percentages (5, 10, and 20) of Zn and Cu doped cerium oxide nanoparticles (CNPs) with a forced hydrolysis method. Following synthesis, various physical, chemical, and biological assays were implemented to investigate the metal-doped CNPs' ability to provide therapeutic ROS modulation. The biological implications of these NPs were studied on MCF-7 breast cancer cells as well as on a healthy endothelial (HUVECs) cell line. The prooxidant characters of the tested metal-doped CNPs formulations were found to lead to cell death, whereas the converse antioxidant character of these NPs may speak to the ideal treatment outcome toward healthy human cells. Based on prior evidence that oxygen vacancies and transition-metal centres may focus more on antioxidant or pro-oxidant behaviour, it was hypothesized that dopant gated catalytic programming can be used to tune nanoceria toward distinct redox regimes, favouring controlled ROS amplification in cancer cells while preserving tolerability in normal vascular cells.^10–13,27,32^

## 2. Experimental

### 2.1 Materials

Copper(ii) nitrate hexahydrate (Cu(NO_3_)_2_·6H_2_O, 99% purity), zinc nitrate hexahydrate (Zn(NO_3_)_2_·6H_2_O, 99% purity), cerium nitrate hexahydrate (Ce(NO_3_)_3_·6H_2_O, 99.999% purity), dimethyl sulfoxide (DMSO), and hydrogen peroxide (H_2_O_2_ 3 w/v%) were purchased from Sigma-Aldrich (Merck KGaA, Milwaukee, WI, USA). Ammonium hydroxide solution (1 N NH_4_OH) used for nanoparticle synthesis was purchased from Alfa Aesar. All chemicals were used as received from the supplier without any further purification. Deionized water (DH_2_O) was used for all nanoparticle synthesis and characterization experiments. For cell studies, the human breast cell (MCF-7, HTB-22) was obtained from the ATCC (American Type Culture Collection). Fetal bovine serum (FBS), Eagle's Minimum Essential Medium (EMEM), penicillin–streptomycin (Pen–Strep), Trypsin–EDTA, and phosphate-buffered saline (PBS), and were purchased from Thermo Fisher Scientific.

### 2.2 Nanoparticle synthesis

**Fig. 1:**
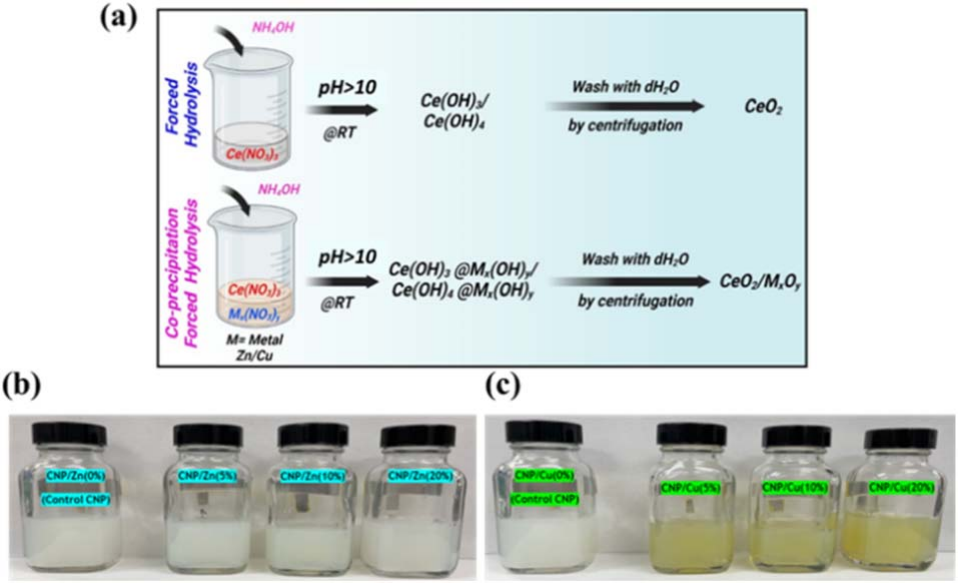
Schematic of the forced hydrolysis synthesis of zinc- and copper-doped CNPs. (a) Particle formulations were synthesized using varying dopant concentrations (0, 5, 10, and 20 mol%) of zinc or copper nitrates relative to cerium nitrate. Forced hydrolysis was induced by base addition under continuous stirring, followed by washing and ultrasonication. Final colloidal suspensions were stabilized by pH adjustment to 3.5 using nitric acid. (b) Zinc-doped CNPs and (c) copper-doped CNPs prepared at corresponding mol% dopant levels.

**Fig. 2:**
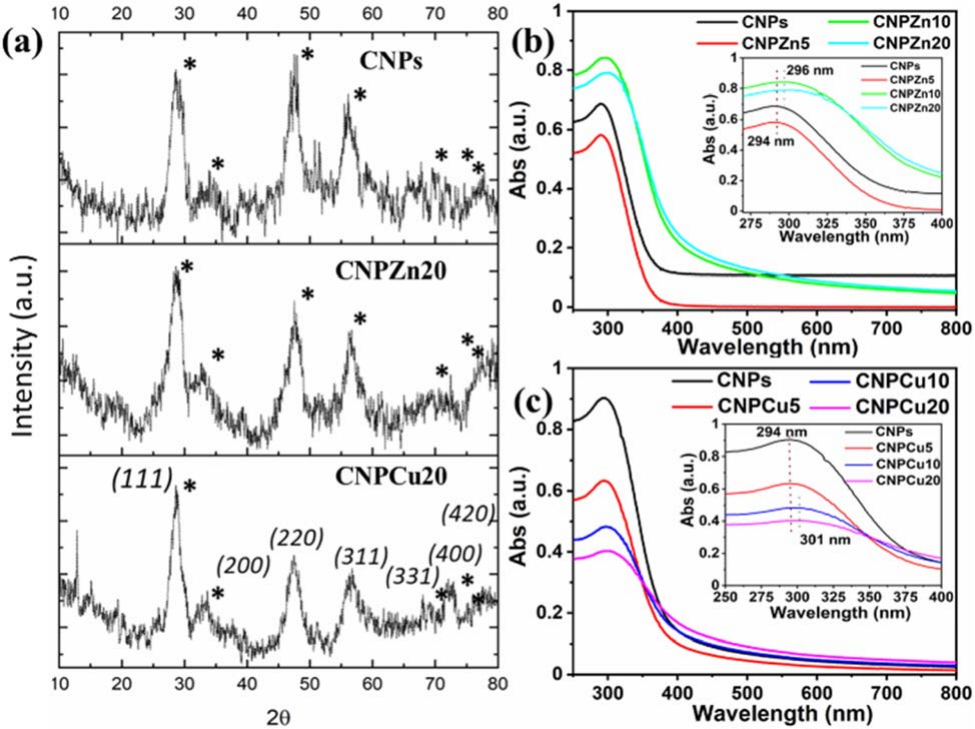
Structural and optical characterization of metal-mediated cerium oxide nanoparticles. (a) X-ray diffraction (XRD) patterns of CNPs, CNPZn20, and CNPCu20 samples. All patterns exhibit diffraction peaks corresponding to the cubic fluorite CeO_2_. The absence of secondary phase reflections and the observed peak broadening in doped samples indicate successful dopant incorporation within the nanocrystalline lattice. (b and c) UV-visible absorption spectra of (b) Zn-doped and (c) Cu-doped CNPs at increasing dopant concentrations (0–20 mol%). Insets show the magnified absorption region (275–400 nm), highlighting concentration-dependent shifts in absorption maxima. Zn-doped CNPs exhibited an absorption peak at approximately 294–296 nm, while Cu-doped CNPs showed a red-shifted absorption at approximately 294–301 nm relative to pristine CNPs, consistent with dopant-induced modification of the electronic structure of the cerium oxide lattice.^44,45^

Zinc- and copper-doped cerium oxide nanoparticles (CNPs) were synthesized at dopant concentrations of 0, 5, 10, and 20 mol% using a forced hydrolysis method. Cerium nitrate hexahydrate (52 mM) was dissolved in 40 mL of DH_2_O under continuous stirring to ensure complete dissolution. For each synthesis, four separate formulations were prepared for zinc and copper doping, respectively. Aqueous solutions of zinc nitrate or copper nitrate corresponding to 0, 5, 10, or 20 mol% relative to cerium nitrate were produced separately by dissolving the precursors in 10 mL of DH_2_O (Fig. 1a). Each dopant solution was then added to the cerium nitrate solution, yielding a total reaction volume of 50 mL. Forced hydrolysis was initiated by the rapid dropwise addition of 5 mL of 1 N ammonium hydroxide (NH_4_OH) under vigorous stirring at 550 rpm. The reaction mixtures were maintained under continuous stirring at room temperature for 5 hours. During the reaction, visible color changes in solution indicated the nanoparticle formation. Following completion of the reaction, the resulting suspensions were centrifuged (12 000 rpm for 10 min). The supernatant was decanted, and the precipitated nanoparticles were washed three times with DH_2_O by repeated resuspension and centrifugation to remove residual ions and unreacted species. After the final wash, the nanoparticle pellets were resuspended in 35 mL of DH_2_O and ultrasonicated to ensure dispersion. The suspensions were allowed to equilibrate overnight. Colloidal stability was achieved by adjusting the suspension pH to approximately 3.5 using nitric acid (HNO_3_) (Fig. 1b and c). The optical properties of the pristine and metal-doped CNPs were further characterized by UV-Vis absorption spectroscopy (Fig. 2b and c). Any remaining aggregation was mitigated by dilution or additional pH adjustment as needed. This procedure was identical for all dopant concentrations. Hereafter, zinc-doped cerium oxide nanoparticles with 0, 5, 10, and 20 mol% Zn were designated as CNPs (undoped), CNPZn5, CNPZn10, and CNPZn20, respectively. Copper-doped cerium oxide nanoparticles with 5, 10, and 20 mol% Cu were designated as CNPCu5, CNPCu10, and CNPCu20.

### 2.3 Characterization

Pure and metal-doped cerium oxide nanoparticles (CNPs) synthesized *via* forced hydrolysis were characterized using UV-Vis, X-ray diffraction (XRD), XPS spectroscopy, and DLS techniques to evaluate optical properties, surface chemistry, oxidation states, particle size and surface potential of the nanoparticles. Superoxide dismutase, catalase and cytotoxicity studies were also performed to determine the anticancer efficacy.

#### 2.3.1 Structural and optical analysis

##### 2.3.1.1 X-ray diffraction (XRD)

Synthesized pure and metal-doped CNPs were analysed by powder X-ray diffraction using Empyrean Panalytical X-ray Diffractometer to determine their crystalline structure. XRD patterns were collected using Cu Kα radiation source (1.5405 Å) over a 2*θ* range of 10–80°, with a step size of 0.02° and scan rate of 2° min^−1^. All diffraction patterns were analysed using XPert High Score analysis software for peak identification and plane indexing. The average crystallite size was approximated through application of the Scherrer equation: *D* = *Kλ*/(*β* cos *θ*); where “*D*” is the crystallite size, “*K*” is the shape factor (0.9), “*λ*” is the X-ray wavelength (Cu Kα, 1.5405 Å), “*β*” (in radians) is the full width at half maximum (FWHM) of the diffraction peak, and “*θ*” is the Bragg angle.

##### 2.3.1.2 UV-visible spectroscopy

UV-vis spectra were collected using a PerkinElmer spectrophotometer over a wavelength range of 220–600 nm to assess structural of ceria and verify the absence of secondary crystalline phases.

##### 2.3.1.3 X-ray photoelectron spectroscopy (XPS)

XPS measurements and analyses were performed to determine material elemental and chemical state compositions. Survey spectra and high-resolution core-level spectra over binding energy regions corresponding to C 1s, O 1s, Ce 3d, Zn 2p, and Cu 2p were acquired for all samples using an ESCALAB 250Xi spectrometer operated at room temperature under ∼1 × 10^−8^ mbar ultrahigh vacuum conditions. A monochromatic Al Kα radiation source (15 kV, 20 mA, 300 W) with a spot size of 600 µm was used. A 30 µL sample of each nanoparticle suspension was drop-cast onto a gold foil and dried under ambient conditions overnight prior to measurement. Binding energies were referenced to the C–C/C–H associated peak at 284.6 eV, attributed to adventitious carbon contamination. Spectral deconvolution was performed using Avantage software with material signal peak fitting and Smart background (Avantage) signal fitting, consistent with established analyses.^19,23,28^ Oxidation-state assignments and peak positions were determined using reference spectra (Thermo Fisher Scientific) and literature values.

##### 2.3.1.4 Dynamic light scattering (DLS)

The surface potential and hydrodynamic particle size of the synthesized nanoparticles were analysed using DLS technique (Malvern Zetasizer Nano instrument). Before analysis, the synthesized nanoparticles were dispersed in DH_2_O at a concentration of 0.5 mg mL^−1^.

##### 2.3.1.5 Transmission electron microscope

The particle size and morphology of pure and metal-doped CNPs were examined using a 300 kV Philips Tecnai HRTEM (high resolution transmission electron microscope). For sample preparation, 10 µL of each nanoparticle's suspension was drop-cast onto separate holey carbon coated copper TEM grids and dried under the infrared light and it's stored under vacuum until analysis.

#### 2.3.2 Enzyme-mimetic activity assays

##### 2.3.2.1 Superoxide dismutase (SOD) activity

Nanoceria are well established to exhibit intrinsic superoxide dismutase- and catalase-mimetic activities arising from reversible Ce^3+^/Ce^4+^ redox cycling at the nanoparticle surface. The superoxide dismutase (SOD)-like activity of pure, zinc, and copper-doped CNPs was evaluated using a commercially available SOD activity assay kit (Dojindo Laboratories), following the manufacturer's protocol. Briefly, 20 µL of each nanoparticle suspension was added to designated sample and blank 2 wells in a Corning Costar 96-well cell culture plate, followed by the addition of 200 µL of water-soluble tetrazolium (WST-1) working solution. Control blanks were prepared according to the supplier instructions to account for background absorbance and reagent contributions, with total reaction volumes kept constant across all wells. After reagent addition, the plate was gently mixed to avoid bubble formation, and 20 µL of enzyme working solution was added to blank and all nanoparticle-containing wells. Absorbance at 450 nm was measured kinetically from 0 to 15 minutes using a multi-well plate reader (FLUOstar Omega, BMG LABTECH). Reaction slopes were determined from linear fits of absorbance *versus* time, and SOD-like activity was calculated as the percentage inhibition rate using the following equation.^22,25^1
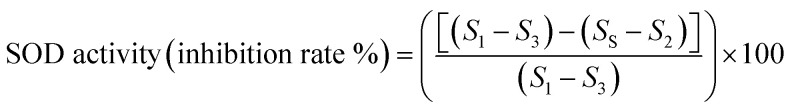
where *S*_1_, *S*_2_, *S*_3_, *S*_S_ are the derived slope values for each of blanks 1, 2, and 3 and synthesized nanoparticles, respectively. Triplicate measurements of each sample as well as all controls blanks were conducted and averaged.

##### 2.3.2.2 Catalase activity

Catalase-like activity was determined using an Amplex Red Hydrogen Peroxide/Peroxidase assay kit (Invitrogen) to quantify residual hydrogen peroxide (H_2_O_2_). Synthesized nanoparticle suspensions (50 µL) were pre-incubated with 50 µL of 8 µM H_2_O_2_ in Corning Costar 96-well cell culture plate for 30 min at room temperature. An Amplex Red working solution containing 0.2 U mL^−1^ horseradish peroxidase (HRP) and 100 µM Amplex Red reagent were prepared according to the manufacturer's instructions and added (100 µL) to each well. Plates were incubated for an additional 30 minutes at room temperature in the dark. Fluorescence intensity of resorufin, proportional to remaining H_2_O_2_ concentration, was measured using an excitation wavelength of 571 nm and an emission wavelength of 585 nm. A standard H_2_O_2_ curve was used to calculate the exact H_2_O_2_ concentration in each synthesized nanoparticle. CAT activity was calculated by the following formula:2
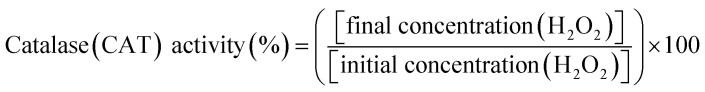


#### 2.3.3 *In vitro* cell studies

##### 2.3.3.1 HUVEC biocompatibility assessment

The biocompatibility of pure, zinc, and copper-doped CNPs was evaluated using human umbilical vein endothelial cells (HUVECs).^22,23,40,41^ HUVECs (ATCC, USA) were grown using commercially available vascular cell basal medium along with endothelial growth factors and maintained at 37 °C in 5% CO_2_ incubator. 0.1% trypsin–EDTA was used to detach the cells and collected using centrifugation (1000 rpm for 5 min) process, and experiments were conducted using cells at passage 3. HUVECs were seeded at a density of 1 × 10^4^ cells per well in 96-well cell culture plates. The plates were incubated for 24 h in CO_2_ incubator to allow cell attachment. After one day, the medium was removed then replaced with fresh medium containing serial dilutions of CNPs, Zn-doped CNPs, or Cu-doped CNPs (7.81–1000 µg mL^−1^). Nanoparticles treated cells were incubated for 1 or 3 days. After the time point, the cell viability was quantified *via* MTT assay. Briefly, 200 µL of culture medium containing 1 mg mL^−1^ of MTT regents was added to each well and incubated for 4 h at 37 °C under 5% CO_2_. After 4 h, MTT solution was removed, followed by 200 µL of DMSO was added to each well to dissolve the formazan crystals. Plates were continuously shaken for 30 min to make complete formazan crystal dissolved and uniform solution. Then, absorbance was measured at 570 nm using a microplate reader (BMG LABTECH). Untreated cells cultured under identical conditions served as a control.

##### 2.3.3.2 MCF-7 anticancer efficacy

The anticancer efficacy of the synthesized nanoparticles was assessed using MCF-7 human breast cancer cells following an identical protocol. MCF-7 cells were seeded at 1 × 10^4^ cells per well in 96-well cell culture plates and it incubated for 24 h to attach the cells on well plate. Cells were then treated with serial dilutions (7.81–1000 µg mL^−1^) of CNPs, Zn, and Cu-doped CNPs and incubated for 1 or 2 days. Cell viability was quantified *via* MTT assay as described above, with untreated MCF-7 cells serving as controls.^10,37^ IC_50_ was defined as the concentration of nanoparticles causing 50% reduction of cell growth as compared to control cells. From MTT results, selectivity index (SI) quantifies the preferential cytotoxicity of nanoparticle formulations toward MCF-7 breast cancer cells relative to normal HUVECs, SI was calculated using below equation.

3 SI = IC_50_(normal cells)/IC_50_(cancer cells)

##### 2.3.3.3 Live/dead Assay

A live and dead fluorescence assay was implemented as a complementary qualitative assessment of cell viability following nanoparticle treatment. HUVECs and MCF-7 cells were treated with pure, zinc-, and copper-doped CNPs at concentrations ranging from 7.81 to 1000 µg mL^−1^. HUVECs and MCF-7 cells were incubated for 1 day prior to staining. Then, the medium was aspirated, and cells were stained by Molecular Probes Live/Dead assay Kit according to the supplier's instructions. A staining solution (mixer of 4 µM ethidium homodimer-1 and 2 µM calcein AM) was added to each well, and the plates were incubated for 30 minutes at 37 °C under 5% CO_2_ in dark conditions. Calcein-positive (live) ware identified by green fluorescence and ethidium homodimer-positive (dead) cells displayed red fluorescence and it imaged through Nikon fluorescence microscope. Representative images were acquired to qualitatively evaluate nanoparticle-induced cytotoxicity and corroborate trends observed in the MTT assay.

### 2.4 Statistical analysis

Catalase, SOD, DLS and Cell studies were performed in triplicate for each condition and average values used for plotting the graph. Statistical analyses were performed using a one-way ANOVA (https://www.sciencedirect.com/topics/food-science/analysis-of-variance) with a Tukey's test for multiple comparisons among experimental groups (GraphPAD Prism, v8, USA). A *p* value < 0.05 was considered statistically significant for all studies. All experiments were performed in quadruplicate and results are reported as mean ± standard error for all biological testing.

## 3. Results

### 3.1 Structural and chemical characterization

#### 3.1.1 X-ray diffraction analysis

X-ray diffraction patterns of undoped CNPs, CNPZn20, and CNPCu20 are presented in Fig. 2a. The undoped CNPs exhibited seven characteristic diffraction peaks indexed to the (111), (200), (220), (311), (331), (400), and (420) crystallographic planes, consistent with the cubic fluorite structure of cerium oxide (ICDD PDF no. 98-026-2755). Both Zn- and Cu-mediated samples retained the same set of fluorite reflections, indicating that incorporation of Zn or Cu did not induce a detectable phase transformation of the ceria lattice.^23,42^ No additional diffraction peaks corresponding to secondary crystalline phases such as CuO, ZnO, or metal hydroxides were observed, suggesting that the dopants are either incorporated within the ceria lattice or present as highly dispersed species below the detection limit of XRD.^20,22,23,42^ However, incorporation of Zn and Cu ions led to a reduction in diffraction peak intensity accompanied by increased peak broadening, indicative of altered crystallinity. The peak broadening implies a decrease in crystallite size upon metal doping. Using the FWHM values for the major crystalline planes of CNPs, CNPZn20, and CNPCu20, the average crystallite size was determined using the Scherrer equation. The estimated crystallite sizes were 6.3 nm for CNPs, 3.4 nm for CNPZn20, and 4.2 nm for CNPCu20. Relative to undoped CNPs, both CNPZn20 and CNPCu20 exhibited reduced diffraction peak intensities and pronounced peak broadening. Peak broadening in doped ceria systems is commonly associated with reduced coherent crystallite size and increased lattice strain arising from dopant incorporation, as reported elsewhere.^23,28,43^ Such structural perturbations are consistent with the ionic radius mismatch between Ce cations and the smaller Zn^2+^ or Cu^2+^ dopants, which can introduce local lattice distortion and promote charge-compensating defect formation.^19,22,23^ Similar suppression of crystallite growth has been reported for Cu- and Zn-doped ceria systems and attributed to dopant-induced inhibition of grain boundary migration during nucleation and growth.^7,12^ Importantly, the preservation of the cubic fluorite phase despite relatively high nominal dopant concentrations (20 mol% of Zn and Cu) highlights the defect tolerance of the ceria lattice, in which charge neutrality is primarily accommodated through the formation of oxygen vacancies rather than phase segregation.^23,42^ The observed reduction in crystallite size concomitantly increases the surface-to-volume ratio, which may influence the density of accessible surface sites relevant to subsequent catalytic interactions.

#### 3.1.2 UV-visible Spectroscopy

UV-vis absorption spectra of undoped and various concentrations of Zn and Cu-doped CNPs are shown in Fig. 2b and c. Undoped CNPs exhibited a broad absorption band spanning approximately 225–350 nm (*λ*_max_ = 294 nm). This absorption peak is characteristic of cerium oxide and is commonly attributed to charge-transfer transitions involving O 2p and Ce 4f electronic states.^19,23^ At low dopant concentrations (5 mol%), no appreciable shift in the absorption maximum was observed for either Zn- or Cu-mediated formulations relative to undoped CNPs. In contrast, samples containing higher dopant concentrations (10 and 20 mol%) exhibited a systematic red shift toward longer wavelengths. For Zn-mediated CNPs, the absorption maximum shifted from 294 nm to approximately 296 nm, while Cu-mediated CNPs exhibited a more pronounced shift to approximately 301 nm at 20 mol% dopant concentration. These shifts are evident in the inset spectra shown in Fig. 2b and c. In addition to spectral shifts, dopant-dependent variations in absorption intensity were observed. Cu-mediated CNPs exhibited a progressive decrease in absorption intensity with increasing Cu content across the measured wavelength range. In contrast, Zn-mediated CNPs displayed higher absorption intensities at increased Zn concentrations, particularly in the near-UV region. No additional absorption bands were detected for any formulation across the 250–600 nm range. The observed red shift in absorption maximum with increasing dopant concentration is consistent with prior reports describing dopant-induced modification of the electronic structure of ceria nanoparticles.^22,23,28,42^ Such shifts have been associated with the introduction of defect-related electronic states within the ceria band structure, arising from aliovalent substitution and charge-compensating defect formation.^20,23^ The more pronounced red shift observed for Cu-mediated CNPs relative to Zn-mediated formulations suggests a stronger perturbation of electronic states in the presence of redox-active copper species, in agreement with previous studies of transition-metal-modified ceria systems.^7,12^ The absence of additional absorption features attributable to CuO or ZnO phases supports the conclusion that the dopants do not form optically distinct secondary phases under the present synthesis conditions. This observation is consistent with the XRD results, which also confirmed retention of the fluorite ceria structure without detectable secondary crystalline phases.

#### 3.1.3 X-ray photoelectron spectroscopy

**Fig. 3:**
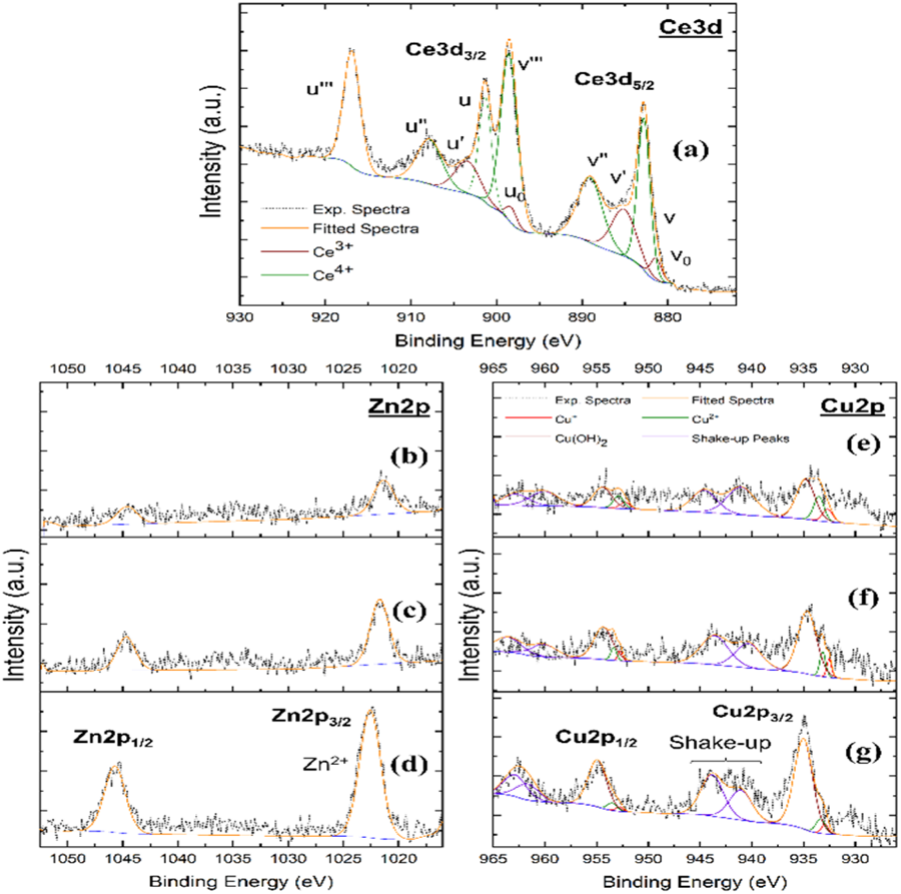
X-ray photoelectron spectroscopy (XPS) analysis of undoped CNPs and Zn- and Cu-doped CNPs. (a) High-resolution Ce 3d XPS spectrum of control ceria nanoparticles (CNPs), displaying the characteristic multiplet structure assigned to Ce^3+^ and Ce^4+^ chemical states. (b–d) Zn 2p spectra of Zn-mediated ceria at increasing dopant concentrations, showing Zn 2p_3/2_ and Zn 2p_1/2_ doublets characteristic of oxidized zinc species. (e–g) Cu 2p spectra of Cu-containing ceria at increasing Cu contents, exhibiting Cu 2p_3/2_ and Cu 2p_1/2_ features accompanied by characteristic shake-up satellite peaks.

X-ray photoelectron spectroscopy (XPS) was employed to confirm the presence of cerium oxide and incorporated metal phases, as well as to determine the associated chemical states, and their relative compositions for all material formulations. High-resolution Ce 3d spectra (Fig. 3a) were deconvoluted into the characteristic spin–orbit components associated with Ce^3+^ and Ce^4+^ species. In accordance with established Ce 3d assignments, *u*_0_, *v*_0_, *u*′, and *v*′ were attributed to Ce^3+^, while *u*, *v*, *u*″, *v*″, *u*‴, and *v*‴ were assigned to Ce^4+^. The surface Ce^3+^ fraction was calculated as:

4 % Ce^3+^ = ((∑∫[*u*_0_, *v*_0_, *u*, *v*]/∑∫[all ceria peaks])) × 100%

Semi-quantitative analysis of the fitted spectra revealed Ce^3+^ surface fractions of approximately 21.1, 12.5, and 18.2 at% for CNPCu5, CNPCu10, and CNPCu20, respectively (Table 1). The zinc-doped CNPs also exhibited an intermediate decrease, with 16.0, 14.3, and 18.2 at% for CNPZn5, CNPZn10, and CNPZn20, respectively (Table 1). The incorporation of Zn and Cu components altered the measured surface Ce^3+^ fraction in a non-linear manner for increasing initial transition metal contents. However, it is evident that transition metal addition influences extent of cerium site reduction and may arise as a consequence of charge imbalances occurring within the forming material as the divalent transition metal species occupy cerium lattice sites.^23,42^ The complex relationship between measured Ce^3+^ fraction and added transition metal content is partly ascribable to the poor transition metal solubility in the ceria lattice and potentially some associative interactions among “dopant” species. Limited incorporation is expected given the appreciable differences in ionic radii, crystal structure, and formal redox states for the transition metals and ceria. The observed relationships between measured Ce^3+^ percentage and initial transition metal content suggest a surface enrichment or segregation that appears to reach a saturation value resulting in ∼18% Ce^3+^. The variability in cerium reduction suggests some association, segregation of the near-surface transition metal-rich phases and/or some kinetic influence. In particular, we note that for both divalent elements, the intermediate concentration of 10% showed the lowest Ce^3+^ content. Further, only CNPCu5 was observed to possess a Ce^3+^ content higher than that of the control, pure ceria formulation (18.2 and 21.1%, respectively). While (near-)surface doping appears to saturate, these sites may also allow subsequent growth of these phases on increasing initial transition metal content. However, XRD analyses showed no appreciable presence of larger transition metal-rich phases; therefore, it is likely that these phases do not grow beyond the size of larger clusters. However, it should be noted that the Ce^3+^ fractions reported here reflect the representative samples prepared in this study; follow-on studies will further and more precisely probe the influence of copper incorporation on cerium reduction and local surface micro-structuring and defect character.

**Table 1:** Physicochemical properties and enzyme-mimetic activities of undoped and Zn- or Cu-doped CNPs. Ce^3+^ surface fractions were determined from XPS analysis. Metal incorporation percentages correspond to nominal dopant concentrations used during synthesis. Hydrodynamic diameters and zeta potentials were measured in aqueous suspension by DLS. SOD and CAT activities are reported as percentage activity relative to control. Values are showed as mean ± standard deviation

Sample code	XPS	DLS		Average particle size from TEM (nm)	SOD activity (%)	Catalase activity (%)
	Ce^3+^ (%)	Hydrodynamic size (d.nm)	Zeta potential (mV)			
CNPs	18.2	89.80 (±3.81)	28.5 (±0.44)	7 ± 2	13.03 ± 1.8	75 ± 2
CNPCu5	21.1	106.7 (±2.56)	23.6 (±0.15)	6 ± 1	22.95 ± 2.8	75 ± 1
CNPCu10	12.5	128.3 (±6.55)	27.9 (±0.84)	5 ± 1	41.64 ± 1.2	75 ± 1
CNPCu20	18.2	149.9 (±2.17)	35.1 (±0.27)	5 ± 1	66.28 ± 1.5	81 ± 3
CNPZn5	16.0	99.46 (±0.82)	28.1 (±2.31)	6 ± 1	53.82 ± 0.8	66 ± 1
CNPZn10	14.3	102.8 (±3.63)	27.4 (±4.56)	5 ± 1	27.19 ± 3.6	68 ± 1
CNPZn20	18.2	110.8 (±1.26)	26.1 (±0.92)	4 ± 1	29.73 ± 0.5	47 ± 2

Formulations produced from zinc precursors evidenced signals within the Zn 2p binding energy region (Fig. 3b–d) with binding energies characteristic of ZnO. The Zn 2p spectral features did not exhibit shake-up satellites, consistent with the closed-shell electronic configuration of Zn^2+^. The absence of zinc phases in XRD measurements, along with XPS spectra analyses, suggests nanomaterial zinc phases exist as near-surface dopants and likely associated with oxygen vacancy. High-resolution Cu 2p spectra (Fig. 3e–g) exhibited prominent Cu 2p_3/2_ and Cu 2p_1/2_ spin-coupled peaks accompanied by shake-up satellite features in the 940–945 eV range, indicative of Cu^2+^ species. Minor contributions consistent with Cu^+^ species were also resolved in the fitted spectra. The coexistence of Cu^2+^ and Cu^+^ surface states has been reported previously for copper-modified ceria systems and is likely to be present as hydroxide phases, given their specific binding energies, near the material surface.^7,12^ Similar to our observations for zinc-containing formulations, the absence of CuO-related crystalline reflections in XRD, together with the XPS analyses, is consistent with copper being present predominantly as surface defect-associated species rather than as a distinct bulk CuO phase (at the nanomaterial surface or as independent secondary particles).^20,22,23,42^ Oxygen vacancies are widely considered to influence the redox behavior of cerium oxide nanoparticles; however, direct vacancy quantification was not performed in the present study. Therefore, the role of oxygen vacancies is discussed here as a plausible mechanistic interpretation rather than a directly measured parameter. However, XPS analysis shows direct evidence of Ce^3+^ sites which may facilitate redox reactions.

#### 3.1.4 Particle size and morphology analysis

**Fig. 4:**
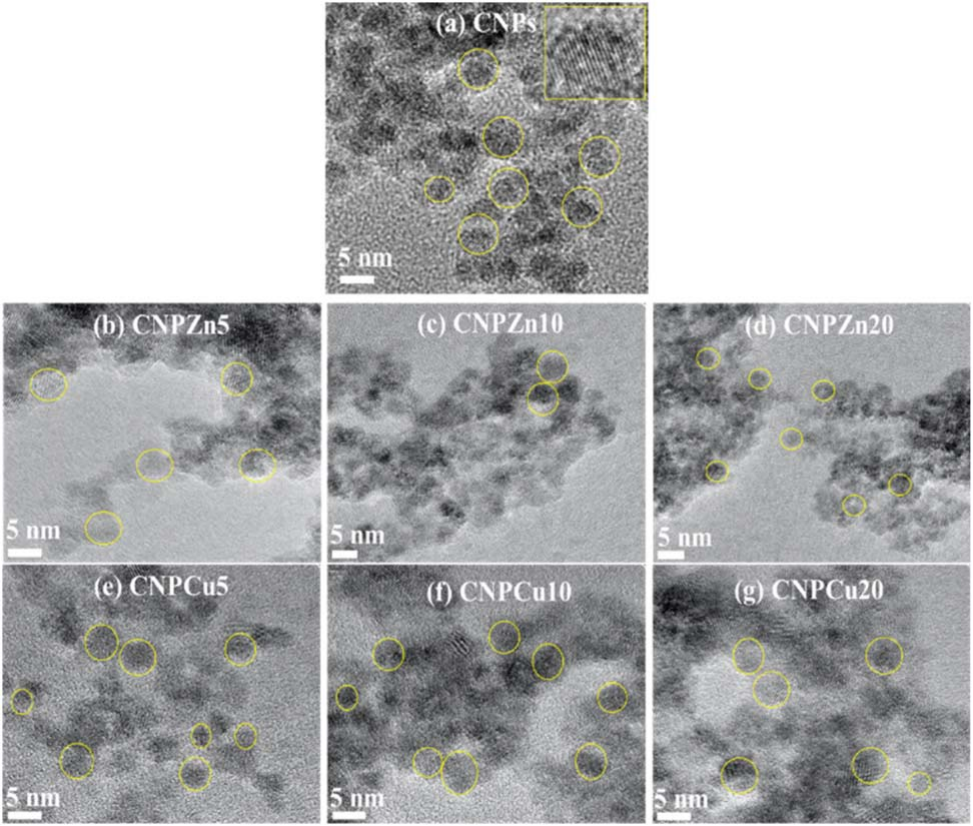
High-resolution transmission electron microscopy (HRTEM) images of zinc- and copper-mediated cerium oxide nanoparticles. (a) Pristine CNPs display well-defined, crystalline primary particles with spherical morphology (yellow circles highlight individual nanocrystallites), and the inset reveals clear lattice fringes characteristic of the fluorite ceria structure. (b–d) Zn-mediated ceria (CNPZn5, CNPZn10, CNPZn20) exhibit densely packed aggregates of primary particles with visible lattice fringes (yellow circles). (e–g) Cu-mediated ceria (CNPCu5, CNPCu10, CNPCu20) display similar primary particle sizes and locally less dense aggregates. The average particle sizes determined from HRTEM analysis were 7 ± 2 nm for pristine CNPs, and 4 ± 1 nm to 6 ± 1 nm for Zn- and Cu-doped formulations (Table 1), indicating that metal dopant incorporation reduces particle size. Scale bars: 5 nm.

Fig. 4a–g presents transmission electron microscopy images of undoped CNPs, Zn- and Cu-doped CNPs. All nanoparticle formulations consisted of predominantly nanosized spherical particles.^46^ The average particle sizes for CNPs, CNPZn5, CNPZn10, CNPZn20, CNPCu5, CNPCu10 and CNPCu20 were 7 ± 2 nm, 6 ± 1 nm, 5 ± 1 nm, 5 ± 1 nm, 6 ± 1 nm, 5 ± 1 nm, and 4 ± 1 nm respectively. Incorporation of higher concentrations of transition-metal (Zn and Cu) dopants led to a gradual reduction in particle size from 7 ± 2 nm to 4 ± 1 nm (Table 1). Similar primary particle sizes across dopant identities suggest that transition metal incorporation does not substantially alter particle morphology and does not result in segregation under the present synthesis conditions.^42,47^ High-resolution TEM images (inset of Fig. 4a) revealed well-defined lattice fringes for undoped CNPs and similar crystalline features observed for all doped formulations. The measured lattice *d*-spacing values ranged from 3.15 to 3.23 Å for all the samples, corresponding to the (111) crystalline plane, in good agreement with XRD results. Although the primary particle sizes were relatively comparable, qualitative differences in aggregation behavior were evident among the formulations. Undoped CNPs displayed a broader distribution of particle shapes and aggregation states, whereas doped samples, particularly at higher Zn (CNPZn20) and Cu (CNPCu20) loadings, showed reduced particle sizes and more aggregates with irregular morphologies. The reduction in particle size observed for CNPZn and CNPCu relative to pristine CNPs is consistent with the role of Zn^2+^ and Cu^2+^ ions as nucleation centers and growth inhibitors during co-precipitation synthesis. The presence of dopant ions in the reaction medium increases the number of available nucleation sites, thereby promoting the formation of a greater number of smaller nuclei. Simultaneously, these ions may adsorb onto the growing nanoparticle surface, restricting further growth and reducing inter-particle aggregation.^48,49^ Such dopant-dependent modulation of nanoceria aggregation behavior and surface energetics has been reported previously for transition-metal-modified ceria systems.^47,50^

These samples were also analysed for hydrodynamic size and surface charge of nanoparticles using dynamic light scattering (DLS) (Fig. S1a–d). Zeta potential measurements (Fig. S1a and b) revealed dopant-dependent differences in colloidal surface charge. Undoped CNPs showed positive zeta potential +28.5 ± 0.44 mV (Table 1). Cu-mediated formulations exhibited slightly decreased zeta potentials for CNPCu5 (+23.6 ± 0.15 mV) followed by a gradual increase in positive zeta potentials with increasing initial Cu content, reaching +35.1 ± 0.27 mV for CNPCu20, a magnitude frequently associated with electrostatically stable dispersions.^47,50^ In contrast, Zn-mediated samples exhibited lower surface potentials (+26.1 ± 0.92 mV to +28.1 ± 2.31 mV), remaining below the commonly cited ±30 mV threshold for strong electrostatic stabilization. These observations indicate that copper mediation enhances surface charge relative to zinc mediation, consistent with prior reports describing dopant-specific surface coordination and hydration effects in ceria-based nanomaterials.^28^ Dynamic light scattering measurements (Fig. S1c and d) revealed substantially larger hydrodynamic diameters relative to primary particle sizes, reflecting the presence of solvated nanoparticle aggregates in aqueous suspension. Undoped CNPs exhibited a mean hydrodynamic diameter of approximately 89.80 ± 3.81 nm (Table 1). Zn-mediated formulations showed a moderate increase in hydrodynamic size, ranging from approximately 99.46 ± 0.82 nm to 110.8 ± 1.26 nm with increasing Zn content. In contrast, Cu-mediated samples exhibited a more pronounced increase in hydrodynamic diameter, reaching approximately 149.9 ± 2.17 nm for CNPCu20. The disparity between TEM-derived primary sizes and DLS-measured hydrodynamic diameters is characteristic of nanoceria systems and is commonly attributed to aggregation and the extent of electrical double-layer volume in solution form.^42,47^

### 3.2 Enzyme-mimetic activity

#### 3.2.1 Superoxide dismutase (SOD) mimetic activity

The catalytic antioxidant potential of the synthesized nanoparticles was analysed by quantifying their catalase^3^ and superoxide dismutase (SOD) activities, which represent sequential steps in detoxifying ROS, with SOD-like activity converting superoxide to hydrogen peroxide and CAT-like activity decomposing hydrogen peroxide to water and oxygen.^14,36^ The SOD-mimetic activity of nanoceria is widely associated with surface Ce^3+^ sites and oxygen vacancies, which provide the redox flexibility required for superoxide reduction.^17,18^ Undoped CNPs in this study exhibited relatively low SOD activity (13.03 ± 1.8%) (Table 1), consistent with their modest surface Ce^3+^ fraction (∼10%) determined by XPS, in agreement with reports that vacancy-poor nanoceria shows limited superoxide scavenging capacity.^17,36^

Both Zn- and Cu-mediated formulations displayed enhanced SOD activity relative to CNPs, but with distinct dopant-dependent trends. Zn-mediated samples showed a non-monotonic response: SOD activity increased sharply to 53.82 ± 0.8% in CNPZn5 and then decreased to 27.19 ± 3.6% and 29.73 ± 0.5% in CNPZn10 and CNPZn20, respectively (Table 1). Similar dose-dependent behaviour has been observed for Zn–Ni dual-doped CeO_2_, where moderate metal incorporation boosts SOD activity but higher loading does not produce proportional gains, likely due to partial coverage of active ceria sites by metal-oxide domains or perturbation of the Ce^3+^/Ce^4+^ equilibrium.^14,36^ In the present system, the decline at higher Zn levels is consistent with the emergence of ZnO-like surface coordination in XPS, which may limit access to underlying Ce-vacancy sites.^33^

Undoped CNPs (Fig. 4a) display well-defined, crystalline primary particles with spherical morphology (yellow circles highlight individual nanocrystallites), and the inset reveals clear lattice fringes characteristic of the fluorite ceria structure (Fig. 4b–d). Zn-mediated ceria (CNPZn5, CNPZn10, CNPZn20) exhibits densely packed aggregates of primary particles (∼5 nm) with visible lattice fringes (yellow circles) (Fig. 4e–g). Cu-mediated ceria (CNPCu5, CNPCu10, CNPCu20) display similar primary particle sizes and locally less dense aggregates by TEM compared with some Zn-containing samples. These qualitative differences in aggregation may reflect, but do not unequivocally prove, the higher positive zeta potential measured for Cu-doped formulations.

**Fig. 5:**
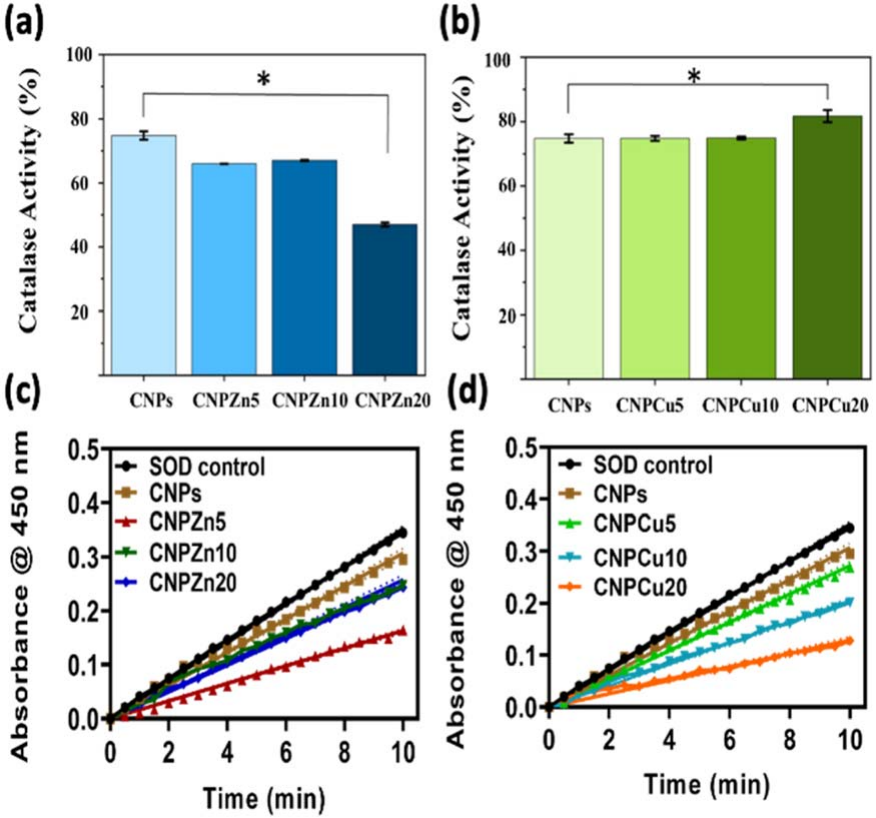
Enzyme-mimetic activity of Zn- and Cu-mediated cerium oxide nanoparticles. (a and b) Catalase-mimetic activity and (c and d) superoxide dismutase (SOD)-mimetic activity of zinc- and copper-mediated cerium oxide nanoparticles synthesized *via* the forced hydrolysis method. Zn-mediated ceria (a) exhibited a concentration-dependent reduction in CAT-like activity relative to undoped ceria, whereas Cu-mediated ceria (b) showed a pronounced increase in CAT-like activity at 20 mol% Cu incorporation. In contrast, SOD-mimetic activity increased with dopant concentration for both systems, with (c) Zn-mediated ceria formulations, showing maximal SOD-like activity at 5 mol% Zn and reduced activity at higher Zn loadings. (d) Cu-mediated ceria formulations, exhibiting a monotonic increase in SOD-like activity up to 20 mol% Cu. Notably, both 20 mol% Zn- and Cu-mediated formulations displayed significantly greater superoxide scavenging activity compared to undoped ceria, indicating dopant-dependent tuning of catalytic bias.

SOD-mimetic activity for Cu-doped formulations increased with Cu content, rising from 22.95 ± 2.8% (CNPCu5) to 41.64 ± 1.2% (CNPCu10) and reaching 66.28 ± 1.5% in CNPCu20, whereas Zn-doped formulations exhibited maximal SOD-like activity at 5 mol% Zn (Fig. 5c and d and Table 1). These trends indicate that dopant identity and concentration modulate SOD-like behaviour, although the relationship between SOD activity and surface Ce^3+^ fraction is not strictly monotonic across all formulations. This trend is broadly consistent with the modest change in surface Ce^3+^ fraction observed for the highest Cu loading (from undoped CNPs to ∼18% Ce^3+^ in CNPCu20; Section 3.1.3), although the Ce^3+^ content varies non-monotonically with Cu concentration.^28,51^ The Ce^3+^ fractions reported here reflect the representative samples prepared in this study; additional batch replication would be valuable for further quantifying synthesis-to-synthesis variability. Multiple studies of Cu-doped CeO_2_ nanozymes report that increasing Cu content raises the Ce^3+^/Ce^4+^ ratio and oxygen-vacancy density, thereby strengthening SOD-like activity in inflammatory and tumor microenvironments.^28,36^ But, in this In addition, Cu directly in redox cycling (Cu^+^/Cu^2+^), providing auxiliary pathways for superoxide conversion that work synergistically with the ceria lattice.^28,48^ The combination of higher Ce^3+^ surface density and additional Cu redox centres likely underpins the superior SOD performance of CNPCu20 relative to both undoped and Zn-mediated formulations.

#### 3.2.2 Catalase mimetic activity

Catalase-mimetic activity in nanoceria is generally attributed to Ce^4+^-rich surface domains that can oxidize H_2_O_2_, with overall efficiency depending on the dynamic Ce^3+^/Ce^4+^ redox cycle.^19,20^ Undoped CNPs displayed high CAT activity (75 ± 2%) (Fig. 5a and b and Table 1), consistent with their Ce-dominated surface chemistry and with prior observations that Ce-rich nanoceria is particularly effective in H_2_O_2_ decomposition.^20^

Zn-mediated samples showed a gradual reduction in CAT activity with increasing Zn content, decreasing from 66 ± 1% (CNPZn5) to 68 ± 1% (CNPZn10) and 47 ± 2% (CNPZn20) (Table 1). A similar trend has been reported for Zn-containing doped ceria, where non-redox-active Zn perturbs the Ce^3+^/Ce^4+^ equilibrium and can diminish peroxide decomposition efficiency at higher dopant loadings.^14,36^ The lower zeta potentials of Zn-rich formulations (+26.1 ± 0.92 mV to 28 ± 2.31 mV) may additionally promote mild aggregation, effectively reducing the reactive surface area available for catalysis, consistent with reports that colloidal destabilization can attenuate enzyme-mimetic performance of nanoceria dispersions.^17^

Cu-mediated samples behaved differently. CNPCu5 and CNPCu10 maintained CAT activities comparable to CNPs (75 ± 1%), while CNPCu20 exhibited the highest CAT activity of 81 ± 3% (Table 1). This simultaneous enhancement of both SOD and CAT activities contrasts with the trade-offs often observed in undoped nanoceria, where conditions that maximize Ce-driven SOD activity can compromise Ce-dependent CAT performance.^19,20^ Cu-doped CeO_2_ nanozymes described in recent studies show analogous dual-enzyme behaviour, in which Cu incorporation not only increases vacancy density but also introduces additional catalytic sites capable of mediating peroxide reactions through Fenton-like or peroxidase-like pathways.^28,51,52^ In particular, Cu–CeO_2_ systems designed for tumour or inflammatory therapy exhibit strengthened peroxidase (POD)-mimetic activity and efficient modulation of local ROS levels, leveraging both Cu and Ce redox couples to transform peroxides.^51^ The elevated CAT activity observed for CNPCu20 is therefore consistent with a cooperative mechanism in which Ce sites, oxygen vacancies, and surface Cu species jointly facilitate efficient H_2_O_2_ decomposition.^36,53^

Collectively, the SOD and CAT data indicate that transition-metal mediation can be used to tune the balance and magnitude of nanoceria's enzyme-mimetic functions in a dopant-specific manner.^17,51,53^ Zn incorporation provides a pronounced but narrow optimum at low doping levels, whereas Cu doping produces a more robust enhancement, with CNPCu20 achieving the highest combined SOD (66.28 ± 1.5%) and CAT (81 ± 3%) activities in this series. These findings align with prior reports that coupling vacancy engineering with redox-active dopants is an effective design strategy for high-performance ceria-based nanozymes^14,36,51^ and position Cu-mediated ceria as a particularly promising platform for applications requiring broad-spectrum ROS regulation. The simultaneous enhancement of both SOD-like and catalase-like activities in the Cu-doped samples suggests that their catalytic behavior may not be explained solely by the conventional Ce^3+/^Ce^4+^dependent trade-off reported for nanoceria, in which SOD- and catalase-like activities depend strongly on the surface redox state.^54,55^ This observation is therefore consistent with the possibility that Cu-related redox mediation also contributes to the overall enzyme-mimetic activity.^32^

### 3.3 Biological studies

#### 3.3.1 Cell viability and cytotoxicity trends

The biological responses of undoped and metal-mediated cerium oxide nanoparticles were evaluated using MTT cell viability assays and qualitative Live/Dead staining in MCF-7 breast cancer cells and normal HUVECs. For the purposes of this study, cell viability values below 80% were operationally defined as indicative of cytotoxic influence under the applied experimental conditions. This threshold is commonly employed in *in vitro* nanotoxicology studies to distinguish biologically relevant reductions in metabolic activity from minor adaptive responses.^19,50^ Furthermore, comparative analysis between normal and cancer cells provides evidence of the selective cytotoxicity of these nanoparticles, demonstrating enhanced effectiveness in inducing cancer cell death while largely preserving normal cell viability.

**Fig. 6:**
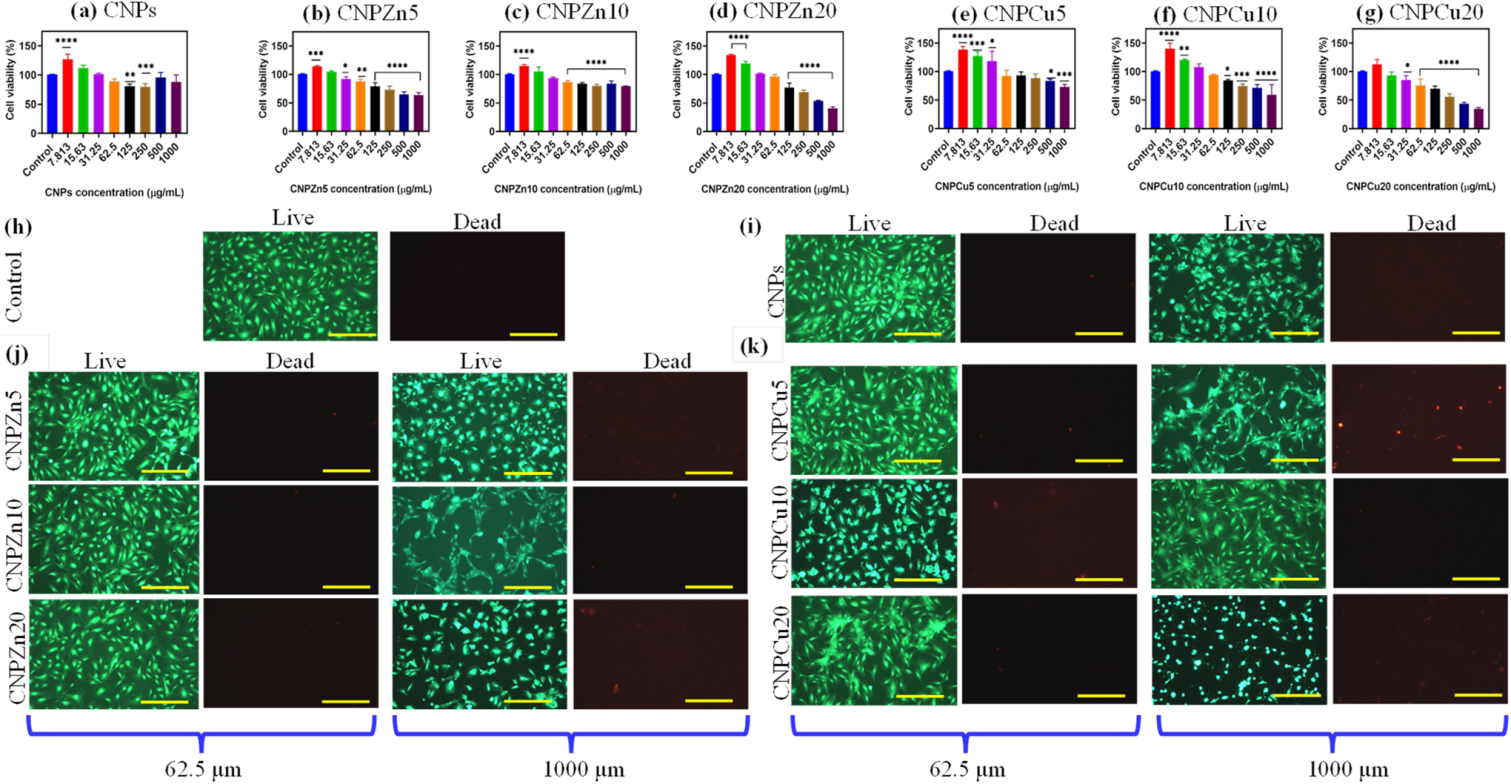
Cell viability and live/dead staining of HUVECs exposed to undoped and metal-mediated cerium oxide nanoparticles. (a–g) MTT cell viability results for HUVECs following 24 h exposure to increasing concentrations (7.81–1000 µg mL^−1^) of (a) undoped CNPs, (b) CNPZn5, (c) CNPZn10, (d) CNPZn20, (e) CNPCu5, (f) CNPCu10, and (g) CNPCu20. Live/dead fluorescence microscopy images of HUVECs following 24 h exposure to, control cells (h), pure CNPs (i), Zn-mediated (j) and Cu-mediated (k) CNPs at selected concentrations (62.5 µg mL^−1^ and 1000 µg mL^−1^). Live cells are stained green and dead cells red. Data are shown as mean ± standard error (*n* = 4). Statistical significance relative to control cells was determined using one-way ANOVA and is denoted by *p < 0.05, **p < 0.01, ***p < 0.001, and ****p < 0.0001. All images were acquired using identical magnification; scale bars correspond to 100 µm.

**Fig. 7:**
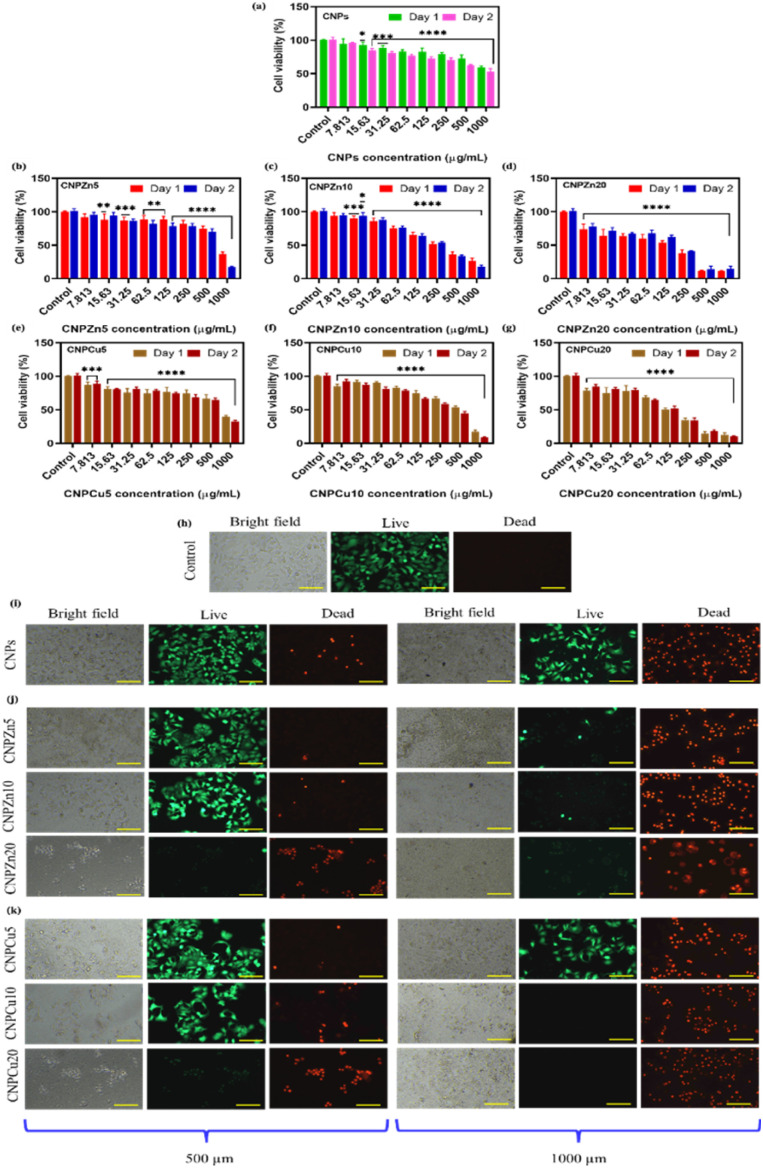
Cytotoxic response of MCF-7 breast cancer cells to undoped and metal-mediated cerium oxide nanoparticles. (a–g) Cell viability assessed by MTT assay following 24 h and 48 h exposure to increasing concentrations (7.81–1000 µg mL^−1^) of undoped CNPs, CNPZn5, CNPZn10, CNPZn20, CNPCu5, CNPCu10, and CNPCu20. Representative bright-field and live/dead fluorescence images of MCF-7 cells following 48 h for control cells and exposure to selected nanoparticle concentrations (500 µg mL^−1^ and 1000 µg mL^−1^) of pure CNPs, Zn- and Cu-mediated CNPs (h–k). Dead cells appear red (ethidium homodimer-1) and live cells are stained green (calcein AM). Data are reported as mean ± standard error (*n* = 4). A statistical significance with respect to the control cell is denoted as follows *p < 0.05, **p < 0.01, ***p < 0.001, and ****p < 0.0001. All images were taken using identical magnification; scale bars correspond to 100 µm.

MTT assays revealed a concentration-dependent decrease in cell viability for CNPs, CNPZn5, 10 and 20 and CNPCu5, 10 and 20 nanoparticles across the tested concentration range (7.81–1000 µg mL^−1^) in HUVECs (Fig. 6a–g). Within the first 24 h of exposure, HUVECs exhibited a slight increase in viability at low concentrations (7.813 µg mL^−1^) followed by no major changes up to 1000 µg mL^−1^ for undoped CNPs (Fig. 6a).^17^ In contrast, Zn, and Cu-mediated CNPs showed approximately 10–20% increase in cell viability at low concentrations (7.813 and 15.63 µg mL^−1^), subsequently, a gradual decline in viability with increasing nanoparticle concentration from 31.25 µg mL^−1^ to 1000 µg mL^−1^ (Fig. 6b–g). These results indicate time- and dose-dependent cytotoxic responses consistent with previous reports for nanoceria systems under oxidative challenge conditions.^22,23,42^ Qualitative live/dead assay was performed on HUVECs exposed to nanoparticles concentration of 62.5 µg mL^−1^ and 1000 µg mL^−1^ for 24 h (Fig. 6h–k). The live/dead staining results corroborated the MTT assay findings, revealing concentration-dependent viability patterns. HUVECs exposed to lower nanoparticle concentrations (≤62.5 µg mL^−1^) predominantly retained viable morphologies (Fig. 6i–k) whereas cells cultured at higher concentrations exhibited reduced viability, as evidence by decreased green fluorescence intensity (Fig. 6i–k). Fig. 7a–d presents the MTT and live/dead assay results for MCF-7 cells cultured with pure, Zn-, and Cu-mediated CNPs nanoparticles for day 1 and day 2. Pure CNPs exhibited a significant reduction in cell viability at higher concentrations (≥62.5 µg mL^−1^). However, even at the highest tested concentration (IC_50_ ≧ 1000 µg mL^−1^), pure CNPs were unable to completely eliminate MCF-7 cells. Additionally, no significant difference in cell viability was observed between day 1 and day 2, suggesting that pure CNPs induce limited cytotoxicity even after prolonged exposure (Fig. 7a). Fig. 7b–d shows the viability of MCF-7 cells exposed to varying concentrations of CNPZn5, CNPZn10 and CNPZn20. The percentage of cell viability gradually decreased with increasing nanoparticle concentration from 7.813 µg mL^−1^ to 1000 µg mL^−1^ for all Zn-doped samples. At day one, the IC_50_ values for CNPZn5, CNPZn10 and CNPZn20 were determined to be 1000 µg mL^−1^, 250 µg mL^−1^ and 62.5 µg mL^−1^, respectively (Fig. 7b–d). Among the tested formulations, CNPZn20 exhibited the strongest cytotoxic effect toward MCF-7 cells, achieving an IC_50_ of 62.5 µg mL^−1^ after one days of exposure. In contrast, undoped CNPs displayed substantially lower cytotoxic potency, with an IC_50_ of approximately 1000 µg mL^−1^ under identical conditions, corresponding to an approximately eightfold difference in effective concentration. At day two, there is no significant change in IC_50_ value for all samples. In addition, a live/dead assay along with brightfield images on MCF-7 cells treated with 500 and 1000 µg mL^−1^ of pure CNPs and Zn-doped CNPs after one day (Fig. 7h–j). The results clearly demonstrated a gradual decrease in viable cells (green luminescence) and a corresponding increase in dead cells (red luminescence) with increasing pure CNPs and Zn incorporation into the CNPs (Fig. 7i and j). Brightfield images also showed that overall cell morphology did not markedly change upon nanoparticle exposure. Similar enhancements in cancer cell cytotoxicity have been reported for Zn-containing ceria systems and are frequently associated with dopant-induced modulation of surface redox behaviour rather than nonspecific particle toxicity.^28,56^ Cu-mediated CNPs exhibited a distinct cell-viability profile (Fig. 7e–g). At 24 h, the IC_50_ values for CNPCu5, CNPCu10, and CNPCu20 were 1000 µg mL^−1^, 250 µg mL^−1^, and 62.5 µg mL^−1^, respectively (Fig. 7b–d). After 48 h of exposure, CNPCu20 remained the most potent formulation, with an IC_50_ of 125 µg mL^−1^, reflecting time-dependent shifts in the viability curves. The cell viability decreased with increase in concentration of CNPCu5 and CNPCu10 and these results concur with Zn-mediated CNPs results. Almost complete cell death was observed at the highest concentration (1000 µg mL^−1^) for these samples. CNPCu20 demonstrated comparatively limited cytotoxicity at low concentrations (≤62.5 µg mL^−1^), while viability decreased more substantially at intermediate concentrations (100–250 µg mL^−1^). Complete loss of MCF-7 cell viability was observed at concentrations above 250 µg mL^−1^. This behaviour contrasts with the sharp low-dose cytotoxicity observed for CNPZn20 and suggests dopant-specific modulation of biological response rather than uniform enhancement of nanoparticle toxicity. Comparable concentration-dependent transitions between antioxidant and pro-oxidant behaviour have been reported for Cu-doped ceria systems in cellular models, reflecting sensitivity to local redox balance and cellular context.^23,50^ To assess relative biological selectivity, cytotoxic responses in MCF-7 cells were compared with HUVECs viability under matched exposure conditions (Table 2). While CNPZn20 exhibited pronounced cytotoxicity toward MCF-7 cells (IC_50_ 62.5 µg mL^−1^), HUVECs viability remained >90% at concentrations up to 62.5 µg mL^−1^, yielding a selectivity index (SI) of 8 under the defined criteria. CNPCu20 demonstrated moderate selectivity (SI: 2), whereas undoped CNPs showed minimal differential effects between cell types (SI: ∼1). These results indicate preferential cytotoxic effects toward cancer cells within a limited concentration window, consistent with previous reports of context-dependent redox behaviour in nanoceria systems.^22,28^ In addition, a live/dead assay with brightfield imaging was performed on MCF-7 cells treated with 500 and 1000 µg mL^−1^ of Cu-mediated CNPs (Fig. 7k). Qualitative live/dead staining corroborated the MTT findings of 500 and 1000 µg mL^−1^ Cu mediated CNPs by revealing concentration-dependent viability patterns (Fig. 7k). In MCF-7 cultures, increasing nanoparticle concentration was associated with elevated proportions of non-viable cells. Such differential responses between malignant and non-malignant cells have been reported previously for redox-active nanoceria and are often attributed to differences in baseline oxidative stress and antioxidant capacity rather than intrinsic nanoparticle selectivity.^22,23,42^

**Table 2:** *In vitro* cytotoxicity and relative selectivity of undoped and metal-mediated cerium oxide nanoparticles toward MCF-7 breast cancer cells compared with normal endothelial cells

Formulation	IC_50_ for MCF-7 (µg mL^−1^), 24 h	HUVECs IC_50_ µg mL^−1^ for 24 h	Selectivity index (SI)^a^
Undoped CNPs	>1000	>1000	∼1.0
CNPZn20	62.5	500	8
CNPCu20	125	250	2

a The selectivity index (SI) from an MTT assay was used to quantify the preferential cytotoxicity of nanoparticles toward cancer cells compared to normal cells. The SI was obtained using the following formula, SI = (IC_50_ (normal cells)/IC_50_ (cancer cells)). The cell viability data for pure, Zn- and Cu-mediated CNPs obtained from HUVECs (normal) and MCF-7 (breast cancer) cells were used for SI calculation. Because the IC_50_ value for pure CNPs could not be determined within the tested concentration range, it was assumed to be >1000 µg mL^−1^. IC_50_ values were calculated from MTT viability results of 24 h nanoparticle exposure under same experimental condition for both cells.

Collectively, these results demonstrate that metal mediation substantially alters the cytotoxic response profile of CNPs in a dopant- and concentration-dependent manner. Zn-mediated nanoceria exhibits strong cytotoxic effects toward MCF-7 cells at low concentrations, whereas Cu-mediated formulations display a broader concentration window with moderated low-dose effects. Importantly, the observed differential responses do not imply absolute cancer specificity but rather reflect context-dependent biological interactions consistent with redox-tuneable nanoceria behaviour reported in the literature.^19,22^

## 4. Discussion

This study investigates the effect of Zn- and Cu-doped on CNPs synthesized *via* forced hydrolysis method at dopant concentration varying from 5 to 20 mol%. During forced hydrolysis using NH_4_OH as the precipitating agent, the initial Ce^3+^ precursor undergoes partial oxidation in the presence of dissolved oxygen, yielding a mixture of Ce^3+^ and Ce^4+^ species in the reaction medium. The Ce^4+^ ions subsequently hydrolyze to form Ce(OH)_4_, which upon thermal treatment converts to CeO_2_. The resulting nanoparticles exhibit a mixed-valence surface, wherein Ce^3+^ is preferentially stabilized at the surface and in oxygen-deficient environments due to the formation of charge-compensating oxygen vacancies.^57–59^

This synthesis method successfully produced fluorite crystal structure CeO_2_ nanoparticles with average particles diameters of approximately 4 ± 1 to 7 ± 2 nm, even in the presence of metal ions during the reaction, as confirmed by XRD and TEM analysis. Incorporation of Zn and Cu into the CNPs was further analyzed by XPS chemical-composition analysis. These results showed that the redox states of Ce^3+^ fraction varied in a non-linear manner with increasing initial concentration of Zn and Cu (Table 1). This result suggests that the addition of Zn and Cu influences the extent of cerium site reduction, likely due to charge imbalances introduced when divalent Zn and Cu ions modify cerium oxide surface properties.^23,42^ Dynamic light scattering measurements further showed that all the conditions synthesized nanoparticles exhibited small hydrodynamic diameters and high positive surface charges, indicating strong colloidal stability. These physicochemical similarities across all nanoparticles substantially reduce confounding contributions from aggregation, charge-driven cellular uptake, significant differences in bio-corona formation/composition, or size-dependent toxicity, allowing biological outcomes to be interpreted primarily in the context of dopant-dependent catalytic activity and redox cycling.^60–63^

The comparable cell viability observed across all nanoparticle formulations at 7.813 µg mL^−1^ is consistent with the established low-toxicity profile of nanoceria at sub-therapeutic concentrations, where the antioxidant and ROS-scavenging properties of cerium oxide are known to be cytoprotective rather than cytotoxic in normal endothelial cells.^64,65^ The cytotoxic response of metal oxide nanoparticles, including cerium oxide-based formulations, is well documented to be dose-dependent, with significant differences in cell viability typically emerging at higher concentrations.^66,67^ Therefore, the data presented at 7.813 µg mL^−1^ should be interpreted as reflecting biocompatibility within this concentration range, while the possibility of dose-dependent cytotoxic divergence between undoped and metal-doped formulations at higher concentrations cannot be excluded and warrants future investigation.

**Fig. 8:**
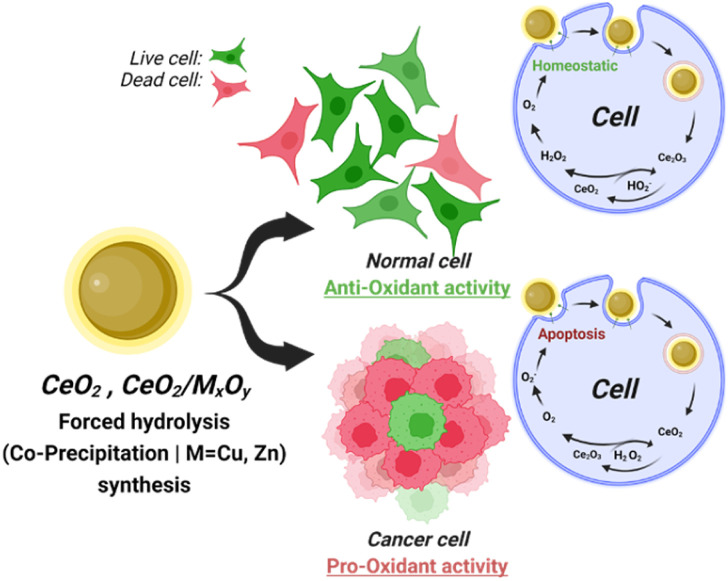
Proposed schematic illustrating context-dependent redox activities of undoped and Zn- or Cu-doped CNPs in normal and cancerous cells. CNPs synthesized *via* forced hydrolysis, with or without Zn or Cu incorporation (CeO_2_, and CeO_2_/M_*x*_O_*y*_), are depicted as exhibiting environment-dependent biological interactions governed by modification in surface chemistry and catalytic activity. In normal endothelial cells (HUVECs), both pure and metal doped CNPs are illustrated as favouring antioxidant or redox-buffering behaviour, with reversible Ce^3+^/Ce^4+^ cycling and oxygen vacancy associated reactivity supporting cellular redox homeostasis and survival (live cell). In breast cancer cells (MCF-7), the same nanoparticles are shown shifting toward pro-oxidant activity under tumour-like conditions, boosting oxidative stress induced apoptosis and loss of cell viability.

Low SOD-like (13.03 ± 1.8%) and high CAT-like (75 ± 2%) activity were observed for the undoped CNPs (Table 1 and Fig. 5a). Collectively, these results exhibit enzyme-mimetic behaviour that is strongly dependent on their surface redox state, where Ce^4+^-rich surfaces promote efficient H_2_O_2_ decomposition while limiting accumulation of long-life period of ROS. Balanced SOD- and CAT-like activity has been proposed to enable nanoceria to buffer oxidative stress by sequential scavenging of superoxide and ROS.^62,63,68^ The high CAT-like activity has for undoped CNPs therefore reflects primarily redox buffering interaction rather than strong oxidative stress. Due to this mechanism, approximately 60% of MCF-7 cells survived at concentration up to 1000 µg mL^−1^ of undoped CNPs. The protection of normal endothelial cell viability across the same concentration range further supports predominantly antioxidant or homeostatic interaction with cellular redox systems. As illustrated in Fig. 8, the antioxidant behavior of CNPs helps to maintain redox homeostatic in the cell environment, thereby supporting proper signaling pathways and protecting cells from oxidative damage. These results are consistent with literature reports that specific nanoceria protect endothelial cells from oxidative stress-induced apoptosis.^62,68–71^

Zn-doped CNPs (CNPZn5) exhibited approximately a 9% reduction in CAT-like and a 40% increase in SOD-like activity compared to undoped CNPs. Furthermore, increasing the initial Zn ion incorporation led to a gradual increase in SOD-like activity (Table 1) while gradually decreasing in CAT-like activity (Table 1, Fig. 5a). This variation represents a catalytic bias,^72,73^ consistent with mechanistic models describing dopants altering the redox behavior of CNPs. Under these conditions, superoxide generated within the cellular environment is efficiently converted to H_2_O_2_, but without a corresponding increase in decomposition of peroxide. Such an imbalance can potentially increase oxidative stress in cells with high basal redox stress, such as many cancer cells^62,63,74^ (Fig. 8). In the case of Zn-doped CNPs, oxidative/redox stress imbalance coincided with the lowest IC_50_ values against MCF-7 cells and the highest apparent *in vitro* cancer selectivity, while endothelial viability remained ≥80% at concentrations up to 62.5 µg mL^−1^. These findings suggest that selective cancer cell vulnerability may arise when peroxide generating reactions are not matched by efficient detoxification, rather than from uniformly elevated redox activity alone. However, Zn-specific effects such as partial Zn^2+^ release, dopant-dependent surface coordination, or altered protein corona composition may also contribute.

Cu-doped CNPs displayed approximately a 6% increase in CAT-like and a 53% increase in SOD-like activity relative to undoped CNPs (Table 1). Increasing the initial Cu ion content produced concurrent enhancement in both SOD- and CAT-like activities (Fig. 5b and d). This dual enhancement aligns with previous reports indicating that Cu-doped CNPs display cooperative redox behavior arising from both Ce^3+^/Ce^4+^ cycling and Cu^+^/Cu^2+^ surface states.^63,68,75,76^ Such behavior indicates an increased overall redox turnover capacity rather than a selective suppression of peroxide removal. In biological systems, Cu doped CNPs have been associated with dose-dependent switching between antioxidant and pro-oxidant effects. Oxidative stress may be buffered at lower concentrations of Cu-doped CNPs but becomes disruptive as catalytic flux increases or under tumour-like conditions. Consistent with this model, Cu-doped CNPs reduced MCF-7 cell viability at lower concentrations than undoped CNPs, however, higher doses were required relative to Zn-doped CNPs to achieve comparable anticancer efficacy, resulting in a lower selectivity index.^62,69,74,76,77^

Direct comparison of Zn- and Cu-doped CNPs indicates that, within these experimental conditions, cancer selectivity correlates more strongly with catalytic imbalance than overall enzyme-mimetic activity. Zn incorporation biased CNPs toward SOD-dominant behavior with reduced CAT-like compensation, whereas Cu incorporation enhanced multiple ROS-processing pathways such as Keap1-Nrf2-ARE, MAPKs, NF-kB, and P13K-Akt, which function simultaneously. This distinction supports the hypothesis that selective cancer cell killing may be favored when superoxide conversion is not coupled to proportional peroxide decomposition, consistent with broader models of tumour-selective nanozyme therapy that exploit imbalanced ROS cascade reactions.^61,62^ Importantly, the mechanistic interpretations presented here are based on correlations between enzyme-mimetic activity assays and cell viability outcomes and do not permit descriptions of intracellular ROS speciation/concentrations, apoptosis signaling processes, or specific cell death pathways (Fig. 8). Particle size also contributes directly to the observed enzyme-mimetic activities directly with respect to the density of reactive, surface vacancy sites, with nanoceria in the ∼4–7 nm diameter range reported to exhibit higher surface Ce^3+^ fractions, increased oxygen vacancy density, and enhanced enzyme-mimetic activity compared with larger particles.^60,63,68,78^

Collectively, these results indicate that undoped CNPs function primarily as a redox-buffering material under the conditions tested, whereas Zn and Cu incorporation alters the surface catalytic behavior in dopant-specific ways that align with differential cytotoxicity in an *in vitro* cancer model relative to healthy endothelial cells. Zn-doped CNPs exhibited the highest apparent cancer selectivity in this system, while Cu-doped CNPs demonstrated concentration-dependent cytotoxic behavior. These findings highlight dopant identity as a controllable design parameter for biasing nanoceria redox activity and reinforce the importance of catalytic balance rather than maximum catalytic activity, in determining biomedical responses. Future studies that directly quantify intracellular oxidant dynamics, metal speciation, and nanoparticle uptake will be essential to resolve the mechanistic basis of this selectivity and to translate these observations into predictive design rules for redox-active nanomedicines.

## 5. Conclusions

This study demonstrates the successful synthesis of nanosized zinc- and copper-doped CNPs using a forced hydrolysis co-precipitation method. Incorporation of Zn and Cu dopants at concentrations up to 20 mol% did not alter the fluorite crystal structure or overall spherical morphology of the CNPs; however, it did modulate key physicochemical properties, including a reduction in primary particle size (from ∼7 to ∼4 nm) and an increase in positive surface charge (from ∼23 to ∼35 mV), accompanied by changes in surface Ce^3+^ content. These results clearly indicate that transition metal doping effectively modulates the functional characteristics of CNPs. Zn doping enhanced SOD-like activity and selectively exploited the elevated basal ROS levels in cancer cells to induce pro-oxidant damage. Cu doping amplified catalase-like activity in our assays and is expected, based on prior work on Cu–CeO_2_ nanozymes, to modulate additional ROS processing activities, thereby enabling tunable oxidative stress responses *in vitro*. *In vitro* cytotoxicity studies revealed that the viability of breast cancer cells (MCF-7) decreased to 50% upon exposure to 62.5 µg mL^−1^ of CNPZn20 and 125 µg mL^−1^ of CNPCu20 for 1 day. Both formulations exhibited minimal toxicity toward normal endothelial cells at the concentrations corresponding to their MCF-7 IC_50_ values (62.5 µg mL^−1^ for CNPZn20 and 125 µg mL^−1^ for CNPCu20), where HUVECs viability remained above 80%, indicating preferential toxicity toward breast cancer cells within this concentration window. The Zn-doped CNPs demonstrated a higher selectivity index (8) compared to Cu-doped CNPs (2), highlighting their superior cancer cell selectivity. Overall, these findings suggest that Zn- and Cu-doped CNPs are promising candidates for selective cancer therapy, offering enhanced anticancer efficacy while minimizing damage to normal cells.

## Author contributions

Samantha Stoltz, Craig J. Neal, Elayaraja Kolanthai, and Yifei Fu, conceptualization, investigation, data curation, methodology, formal analysis, project administration, and writing- original draft. Sudipta Seal – resources, writing – review & editing.

## Conflicts of interest

There are no conflicts to declare.

## Supplementary Material

RA-016-D6RA02316A-s001

## Data Availability

The datasets supporting the findings of this study are available within the article and its supplementary information (SI) files. Supplementary information: Fig. S1: size and zeta potential of Zn and Cu-doped CNPs. See DOI: https://doi.org/10.1039/d6ra02316a.
