# Supplementary material for: Biomedical utility of zinc and copper mediated cerium oxide nanoparticles
Source: RSC Adv. 2026 Apr 30;16(25):22544–58. doi: 10.1039/d6ra02316a (PMC13130048; doi:10.1039/d6ra02316a)
Supplement: RA-016-D6RA02316A-s001 [file RA-016-D6RA02316A-s001.pdf]

Supplementary Information (SI) for RSC Advances.
This journal is © The Royal Society of Chemistry 2026

Biomedical Utility of Zinc and Copper Mediated Cerium Oxide Nanoparticles

Samantha Stoltz[a], Craig J. Neal[a], Elayaraja Kolanthai[a], Yifei Fu[a], Sudipta Seal[a,*]

[a.] *Advanced Materials Processing and Analysis Center, Dept. of Materials Science and Engineering, University of Central Florida, Orlando, Florida, USA. *__Corresponding author:__ Sudipta.seal@ucf.edu*

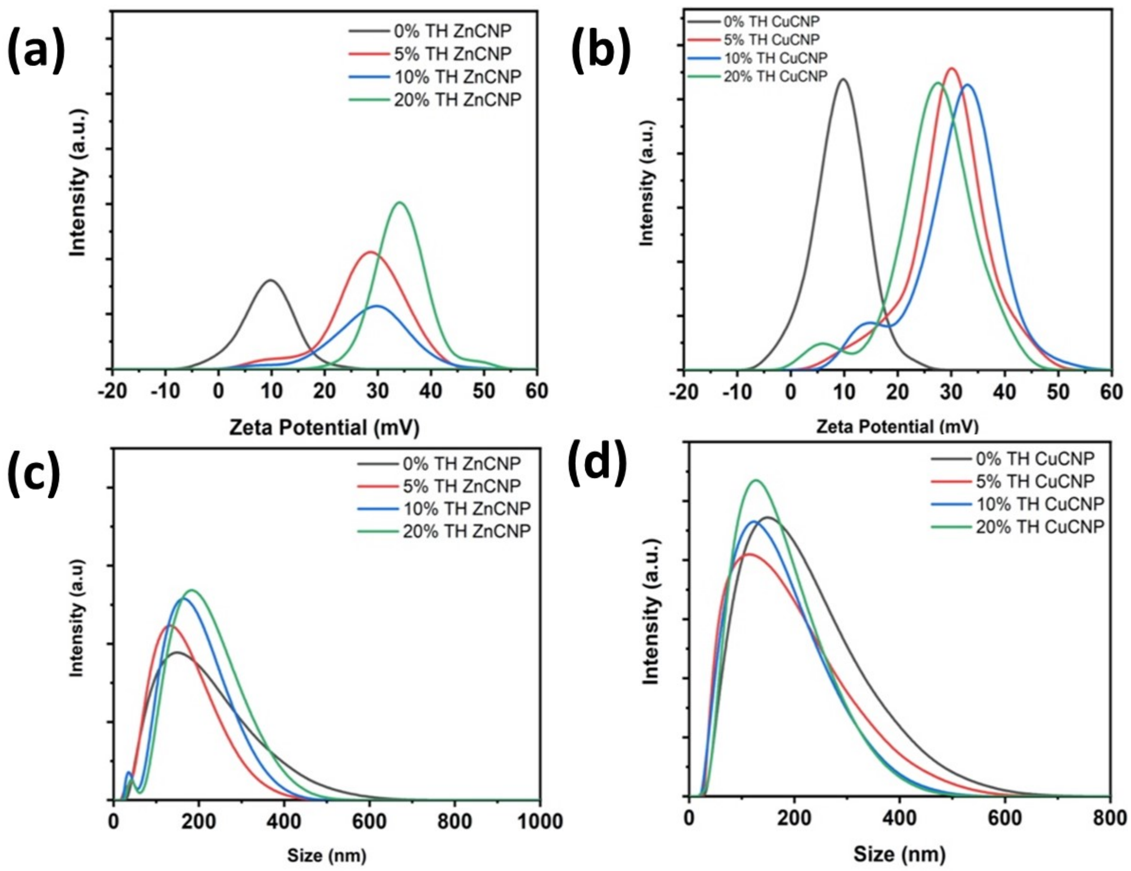

Fig. S1 Size and Zeta Potential of Zn and Cu-doped CNPs. There is an obvious increase in charge for both the Zinc (a) and Copper (b) mediated nanoparticle solutions in comparison with control CNPs. As for size, both the Zinc (c) and Copper (d) decorated Ceria nanoparticle solutions, in addition to the pure Ceria, had significant peaks at 200 nm. This is within range of proper nanoparticle size. Peak broadening suggests discrepancy in particle size, but the absence of a second peak reassures colloid uniformity and stability.
